# IMPlementation of A Relatives’ Toolkit (IMPART study): an iterative case study to identify key factors impacting on the implementation of a web-based supported self-management intervention for relatives of people with psychosis or bipolar experiences in a National Health Service: a study protocol

**DOI:** 10.1186/s13012-017-0687-4

**Published:** 2017-12-28

**Authors:** Fiona Lobban, Victoria Appleton, Duncan Appelbe, Johanna Barraclough, Julie Bowland, Naomi R Fisher, Sheena Foster, Sonia Johnson, Elizabeth Lewis, Céu Mateus, Barbara Mezes, Elizabeth Murray, Puffin O’Hanlon, Vanessa Pinfold, Jo Rycroft-Malone, Ron Siddle, Jo Smith, Chris J. Sutton, Andrew Walker, Steven H. Jones

**Affiliations:** 1 Spectrum Centre for Mental Health Research, Division of Health Research, Faculty of Health and Medicine, Lancaster University, Bailrigg, Lancaster, LA1 4YW UK; 2 Clinical Trials Research Centre, Department of Biostatistics, University of Liverpool, Block F, Waterhouse Bld 1-5 Brownlow Street, Liverpool, L69 3GL UK; 3 Doctorate in Clinical Psychology Programme, Furness College, Lancaster University, Lancaster, LA1 4YG UK; 4 Division of Psychiatry, University College London, 6th Floor, Maple House, London, W1T 7BN UK; 5 Division of Health Research, Lancaster University Furness College, Lancaster University, Bailrigg, Lancaster, LA1 4YW UK; 6 Research Department of Primary Care and Population Health, University College London, Upper floor 3, Royal Free Hospital, Rowland Hill Street, London, NW3 2PF UK; 7 Research Department of Clinical, Educational and Health Psychology, University College London, Gower Street, London, WC1E 6BT UK; 8 The McPin Foundation, 32-36 Loman Street, London, SE1 0EH UK; 9 Bangor Institute for Health & Medical Research, School of Healthcare Sciences, Bangor University, College Road, Bangor, LL572DG UK; 10 Cumbria Partnership NHS Foundation Trust, Penrith, UK; 11 Institute of Health and Society, University of Worcester, Henwick Grove, Worcester, WR2 6AJ UK; 12 Lancashire Clinical Trials Unit, Faculty of Health and Wellbeing, University of Central Lancashire, Preston, PR1 2HE UK

**Keywords:** Implementation, Case series, Early intervention services, Mental health, Relatives, Web-based, Digital psychosis, Bipolar

## Abstract

**Background:**

Web-based interventions to support people to manage long-term health conditions are available and effective but rarely used in clinical services. The aim of this study is to identify critical factors impacting on the implementation of an online supported self-management intervention for relatives of people with recent onset psychosis or bipolar disorder into routine clinical care and to use this information to inform an implementation plan to facilitate widespread use and inform wider implementation of digital health interventions.

**Methods:**

A multiple case study design within six early intervention in psychosis (EIP) services in England, will be used to test and refine theory-driven hypotheses about factors impacting on implementation of the Relatives’ Education And Coping Toolkit (REACT). Qualitative data including behavioural observation, document analysis, and in-depth interviews collected in the first two EIP services (wave 1) and analysed using framework analysis, combined with quantitative data describing levels of use by staff and relatives and impact on relatives’ distress and wellbeing, will be used to identify factors impacting on implementation. Consultation via stakeholder workshops with staff and relatives and co-facilitated by relatives in the research team will inform development of an implementation plan to address these factors, which will be evaluated and refined in the four subsequent EIP services in waves 2 and 3. Transferability of the implementation plan to non-participating services will be explored.

**Discussion:**

Observation of implementation in a real world clinical setting, across carefully sampled services, in real time provides a unique opportunity to understand factors impacting on implementation likely to be generalizable to other web-based interventions, as well as informing further development of implementation theories. However, there are inherent challenges in investigating implementation without influencing the process under observation. We outline our strategies to ensure our design is transparent, flexible, and responsive to the timescales and activities happening within each service whilst also meeting the aims of the project.

**Trial registration:**

ISCTRN 16267685 (09/03/2016).

## Background

“Going Live With a Health IT System is the Beginning, Not the End” [1]

### Context

Across the world, there is a growing interest in the application of self-management approaches as a clinical and cost-effective way to empower people to manage long-term health conditions. A lot of money is spent on developing and testing new health technologies, but there is little understanding of how they can be successfully implemented in routine clinical practice [2]. This is particularly true for digital health interventions, the development of which has seen a huge explosion in recent years, but the majority of which have yet to be successfully integrated into routine clinical practice.

Governing bodies around the world recognise the benefits and cost savings that can be achieved through harnessing digital technology within clinical settings [3, 4]. Not only can such technology offer a possible solution to address the increasing demand for ever more sophisticated and available healthcare in wealthier nations, but also an invaluable opportunity to improve healthcare delivery in developing nations, where it is estimated that more people have access to mobile phones than to water or sanitation [5, 6]. However, digital interventions present particular challenges to healthcare providers, evident by expensive information technology (IT) failures, most notably the UK National Programme for IT in the National Health Service (NHS) which cost approximately 10 billion pounds and failed to deliver its key aims [7–10], and more recently, global privacy concerns linked to data breaches [11]. In developing countries, additional challenges include unreliable web and mobile access, lack of technical expertise and infrastructure, and illiteracy [5]. Some of these challenges are linked to the technology itself, but the greatest challenges are in transforming the ways in which people think about delivery of healthcare and developing new ways of working within healthcare systems [12]. Whilst it is perhaps inevitable that such transformation will be difficult and costly, we need to learn from each attempt to better understand the factors that underlie successful implementation.

### Web-based interventions

There is growing evidence for short-term benefits of web-based psychological treatments for depression and anxiety disorders compared with waitlist (WL) controls [13, 14]. Online interventions are being rapidly developed for psychosis and bipolar disorder, where data supports their feasibility and acceptability [15–18]. Web-based platforms are particularly suited to delivering standardised psychoeducation and facilitating peer support through online forums. Online support may be particularly useful for relatives of people with chronic health conditions [19], due to the flexibility of use and empathy and support from being linked to other carers sharing similar concerns and difficulties [20].

Although some web-based platforms and mobile Apps are designed to be used as standalone interventions by the general public, many are designed as part of broader packages of clinical care and often require staff support to engage people in their use. Thus, implementation of digital interventions is complex, often requiring behaviour change at many levels of the healthcare system. Whilst the importance of organisational factors such as management structures and adequate resources is well recognised, there is a need for more in-depth understanding of how both clinical staff and service users make sense of any new intervention, appraise its worth, engage with it, and monitor its ongoing usefulness [21]. Ideally, these considerations inform the development and design of the intervention from the very outset, but even where this occurs, it may still be necessary to develop of an implementation intervention to facilitate use of the digital intervention within the specific context in which it is being delivered [22]. Attempts to deliver web-based mental health interventions such as Beating the Blues at scale in the UK [23], and as part of routine care in the US [24], have highlighted great difficulties in getting patients to use them or staff to integrate them into practice.

In this study, we aim to understand the key factors impacting on the implementation of the Relatives’ Education And Coping Toolkit (REACT) in UK mental health services and use this understanding to develop an implementation plan. By drawing on implementation theory, we will highlight what can be learnt that is of value to implementation of other digital interventions in different contexts. REACT is a supported self-management toolkit which offers easily accessible evidence-based information and support for relatives of people with psychosis or bipolar disorder. Relatives of people with severe mental health problems provide the vast majority of care, saving the NHS an estimated £1.24bn per year in the UK [25], but this is associated with high levels of distress in relatives [26, 27], significant practical, financial, and emotional burden [28]; stigma; worry; shame and guilt [29]; trauma [30]; and loss [31, 32]. REACT has been shown to be effective in reducing distress and improving perceived support and ability to cope in relatives of people with psychosis in early intervention services [33]. Originally offered in paper form and supported by clinical staff on telephone or email, REACT has since been developed to be available online, broadened to include relatives of people with bipolar disorder, and supported online by REACT supporters who may be either peer relatives or clinicians [34]. REACT is designed to meet the NICE (The National Institute for Health and Care Excellence) recommended quality standard in relation to the provision of carer education and support but is not intended to replace more intensive face to face structured family intervention.

### Theoretical framework

There are several models, frameworks, and theories we could have used to guide our work and ensure our findings are interpreted within a theoretical framework that can support their generalisability. We chose to work with normalisation process theory (NPT) as this facilitates generation of specific hypotheses about the process by which a complex healthcare intervention is implemented, embedded, and integrated (or not); can be tested empirically; and has previously been applied in eHealth settings [21, 35, 36].

NPT began as a model (NPM) of the factors that promote or inhibit the routine work of embedding a new health technology into practice. The key constructs identified were interactional workability, relational integration, skill-set workability, and contextual integration. The model has since been developed into a theory which includes the NPM as constituting “collective action” and adds concepts of “coherence” (how actors make sense of a set of practices), “cognitive participation” (the means by which they participate in them), and “reflexive monitoring” (how these practice are then appraised) [37].

## Methods

The aim of this study is to identify critical factors impacting on the implementation of an online supported self-management intervention for relatives of people with recent onset psychosis or bipolar disorder into routine clinical care and to use this information to inform an implementation plan to facilitate widespread use and inform wider implementation of digital health interventions. Similar web-based toolkits are available across a range of mental and physical health problems [38–41]. Whilst some of the challenges of implementation may be highly context specific, it is likely that a detailed, theoretically informed, and contextually rich analysis of the implementation of a digital health intervention into routine clinical practice will highlight important transferable lessons. This study protocol for Implementation of a Relatives’ Toolkit (IMPART) is guided by the Standards for Reporting Implementation Studies (StaRI) statement [42].

Objectives are to:

(i) Measure the uptake and use of REACT by NHS EIP teams and relatives.
(ii) Identify critical factors impacting on implementation of REACT.
(iii) Identify resources required (and cost implications) for successful implementation of REACT in EIP teams.
(iv) Investigate the impact of REACT delivered by EIP teams on self-reported relatives’ outcomes.
(v) Develop a user-friendly REACT implementation plan and related resources to facilitate widespread use and dissemination.
(vi) Use the findings from this study to further develop theories of implementation of digital interventions in real world practice.

### Design

Our research employs a theory-driven multiple case study design [43] using a mixed methods approach integrating quantitative assessments of outcome (delivery and use of REACT) and qualitative assessments of mechanisms including observation, document analysis, and in-depth interviews. Our case is defined as the EIP service in NHS Mental Health Trusts, which are public sector organisations in England that provide early intervention support to people with early signs of psychosis and/or other severe mental health problems (including bipolar disorder) in a particular geographical locality. Depending on the size of the NHS Trust, there are often further embedded units which are locality teams delivering care to distinct geographically defined areas. Data will also be analysed within stakeholder groups, so we can understand the implementation factors impacting on both staff delivering the intervention and relatives receiving it. This will allow us to understand the process of implementation of REACT within a real-world setting and to identify the causal factors impacting on this process. We will first outline a programme theory about the factors we consider will influence successful implementation of REACT. This theory will be based on NPT and facilitated by tools available on the NPT website which can guide this process (www.normalizationprocess.org/). We will generate hypotheses about the mechanisms that will lead to successful outcomes and then test and refine these hypotheses using a series of case studies in which data collected in early cases is used to inform development of our implementation plan and tested in subsequent cases. The first version of our implementation plan (IPv1) will be based on our programme theory. Detailed case study data from two of the participating Trusts (wave 1) will then be used to develop a revised implementation plan (IPv2), and a refinement of our implementation hypotheses. These hypotheses will then be tested using the revised IPv2 in 2 further Trusts (wave 2). Data collection and analysis across these two Trusts will then focus on further hypothesis testing, leading to a further iteration of the implementation plan (IPv3). This will be introduced to the final two Trusts (wave 3), and data from these Trusts will inform the final implementation plan (IPv4).

Case studies can provide rich data and are particularly useful when trying to understand the implementation of a complex intervention in a real-world setting in which the process or context cannot be controlled. REACT is a “complex intervention” [44] because it depends on the actions of individuals, across different contexts, and adapting their behaviour over time. It also produces multiple outcomes which need to be understood. Implementation is made more complex by the context in which the intervention is situated, which is dynamic and includes competing demands on the system. A mixed methods approach including quantitative assessments of outcome (use and impact of REACT) and qualitative assessments of mechanisms including behavioural observation, document analysis, and in-depth interviews is therefore required to attempt to understand this complexity. We have also designed the study to have extensive input from stakeholder groups at each of the Trusts to ensure that the implementation plan is collaboratively designed.

**Fig. 1:**
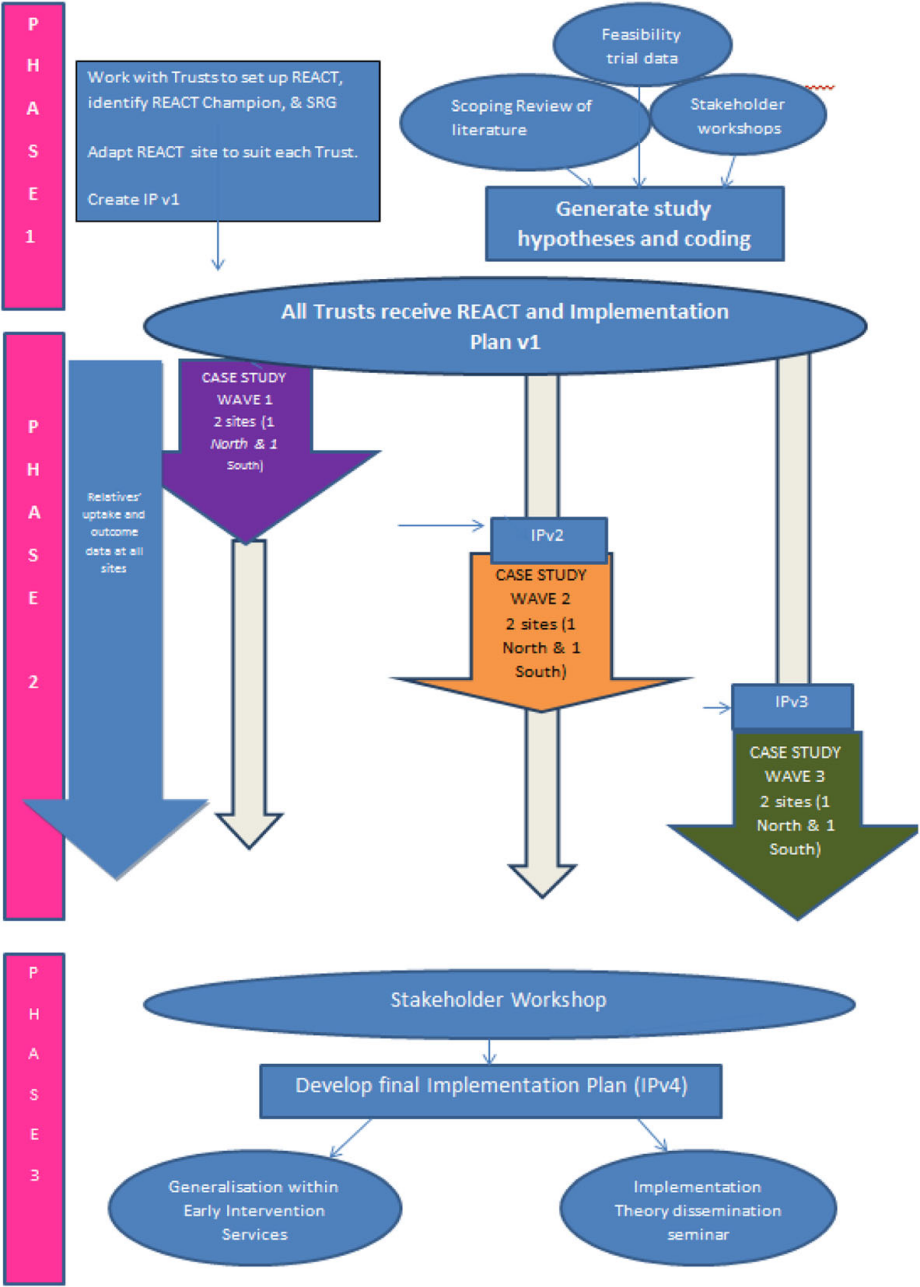
Flowchart showing design of IMPART study

We will use this design flexibly, adapting our approach in response to activity on the ground. Whilst maintaining a focus on specific Trusts at each time point, we will maintain good links with all Trusts and listen to what is happening at each. This ensures that we can respond to significant relevant events at all Trusts as they occur and ensure we are able to collect data where and when it is most informative. This design is shown in Fig. 1 below.

### Setting

EIP teams represent a highly accessible and universal point of access to mental health services for people experiencing first episode psychosis through a range of referral routes including NHS, voluntary sector, and self-referral. They support service users and their relatives. EIP teams were set up in many countries across the world in response to evidence of a “critical period” during the first 3 years of illness during which intervention is thought to be particularly effective in preventing longer term disability [45]. Most teams work with people who have developed symptoms of psychotic illness for the first time, for up to 3 years following first contact (exact criteria vary between services). In the UK, EIP teams generally consist of a mix of psychiatrists, psychologists, care coordinators (social workers, community psychiatric nurses, occupational therapists), and support workers, and aim to deliver services that are consistent with the NICE guidelines for good clinical care [46]. Despite a worldwide recognition of the need for parity of esteem for mental and physical health [47], EIP services have not been immune from the funding challenges faced by all mental health services in the UK [48]. NICE guidelines state that relatives of people with psychosis and bipolar disorder should be provided with information and support and offered structured family intervention to enhance family coping and communication [49, 50]. However, a recent national audit of EIP teams in England showed poor implementation: only 50% of relatives receiving a carer-focussed education and support programme, only 31% offered structured family intervention, and only 12% receiving it [51]. To facilitate implementation of NICE guidelines, the recent “guidance to support the introduction of access and waiting times standards for mental health services in 2015/2016” [52, 53] commits NHS Trusts to ensuring more than 50% of patients experiencing their first episode of psychosis will access NICE concordant care within 2 weeks of referral. Offering REACT should help services to meet the NICE quality standard (NICE QS80) recommendation to offer carers access to an education and support programme, which may therefore facilitate Trust participation and engagement with the IMPART study.

### Sites and participants

The study will be conducted across two regions (North and South of England), with three Trusts participating from each. A formal power calculation is not appropriate for this design, but careful purposive sampling of Trusts will provide sufficient variability to ensure a widely applicable implementation plan. Participating Trusts have been chosen to reflect a range of population size, urbanity, and ethnicity, increasing theoretical generalisability of findings across NHS Trusts. Staff members within the EIP services will be able to invite any relatives of people within this service to use REACT. A sample of staff and relatives in each Trust will be interviewed to understand their experiences of REACT. We will use role within the Trust and levels of engagement with REACT to select participants who can provide a broad range of views.

### Intervention

#### Clinical intervention—REACT

REACT is a comprehensive online recovery-focussed toolkit for relatives of people with psychosis and/or bipolar disorder. It includes online support from REACT supporters (members of the clinical team) via confidential direct messaging and from other relatives through a restricted access forum moderated by the REACT supporters. Trusts can identify the most appropriate supporters, based on available staff resources and structure. However, we have designed the support to be offered by a non-professional support worker (or equivalent) currently working in an EIP team, as it does not require highly trained health professionals, but does require experience in supporting psychosocial interventions, availability, and flexibility. Importantly, support workers are also relatively inexpensive thereby reducing cost barriers to further implementation. REACT supporters will be trained to use REACT using standardised training materials provided online by the research team as part of our initial implementation plan.

**Fig. 2:**
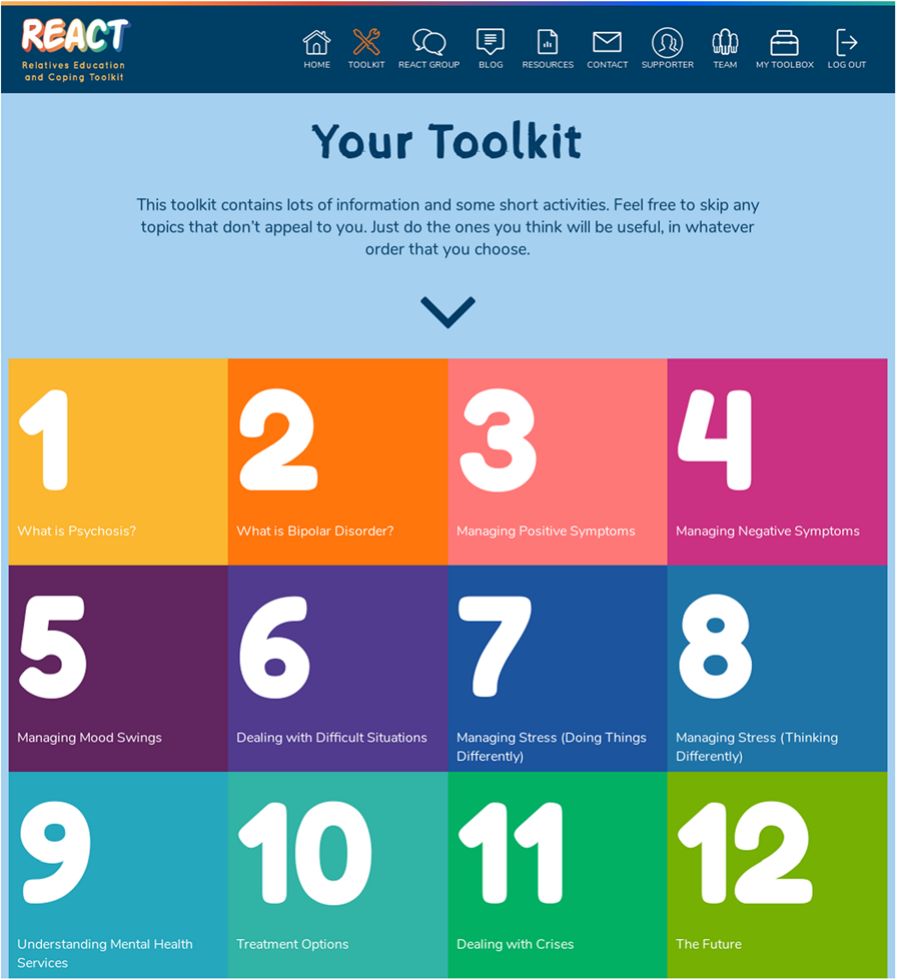
Screenshot of the REACT toolkit homepage, outlining the key modules and features

REACT contains 12 key modules, each of which consists of the following: high-quality standardised written information, videos of clinical experts or experts by experience sharing their knowledge and experiences to illustrate key points, and self-reflection tasks to ensure content is personalised to the user. All videos of relatives telling their real story were retold by actors to preserve anonymity. Figure 2 shows a screenshot of the home page which outlines the modules. A full description of each is given elsewhere [34].

REACT also includes a resource directory that signposts users to a wide range of relevant national and local resources. A “meet the team” page ensures relatives are fully informed about who is delivering the content of the site. Logos for the NHS Trust, Lancaster University, Lancashire Care NHS Trust, UCL, Liverpool Clinical Trials Research Centre (CTRC), and the McPin Foundation are prominently displayed on the login page. “Mytoolbox” offers user a confidential space to save links to any information sources they may want to access easily in future including specific content within the toolkit, their self-reflection tasks, and external web links. A blog page offers a flexible space for additional communication with site users which can be edited by the REACT supporters.

Each Trust can edit some elements of the toolkit to allow limited local adaptation and tailoring of information to a particular organisation (see Table 1).

**Table 1:** Elements of the toolkit each Trust can edit

Editable function	Description
Logo	
Meet the team	Introduces the Trust staff including the IMPART lead and REACT supporters
Emergency contact information	REACT is not a crisis intervention and so directs relatives to appropriate crisis support
Availability and contact e-mails for REACT supporters	Hours available and how often the forum and direct messages are checked to manage expectation
Resources directory	Staff can edit the content to ensure local knowledge is captured and shared
Forum welcome message, rules of use, and suggested topic areas for discussion	This is an opportunity to introduce the forums and mention any particular rules, monitoring times, or anything else appropriate.

#### Implementation intervention

The initial implementation plan (IPv1) will be based on our programme theory and developed during Phase 1 of the study. It will be further developed and tested during Phase 2 as we learn more about specific factors impacting on implementation of REACT. An IMPART lead will be identified in each Trust. They will be a senior member of the clinical service, and their role will be to facilitate stakeholder collaboration, data collection, and implementation of the implementation plan within each case.

### Procedure

#### Phase 1—hypothesis generation

We will outline an implementation theory identifying the factors we think will influence successful implementation of REACT and how they will lead to successful outcome. Our theory will be informed by NPT and specific hypotheses will be further informed by:

1. A systematic review of relevant implementation studies of NHS web-based interventions for people with mental health problems and/or their relatives;
2. Qualitative analysis of data relevant to implementation from the REACT feasibility trial [33] including interviews with EIP staff who have worked as REACT supporters and relatives who used REACT;
3. Stakeholder workshops including staff and relatives at each participating Trust;
4. Synthesis of this data informed by our clinical and theoretical expertise in this area

#### Phase 2—hypothesis testing

We will use a case study design to test our hypotheses about which factors will influence implementation of REACT.

All trusts will be provided with the REACT toolkit and initial implementation plan (IPv1). The exact form and content of IPv1 will depend on findings from Phase 1, but will include an online “how to” manual providing detailed instructions outlining roles and responsibilities for key staff involved in implementing REACT including Trust impart leads, clinical staff who can invite relatives to use REACT, and REACT supporters. Guidance to relatives about using REACT is already embedded within the toolkit.

Detailed case study data (specified below) will be collected and analysed to test our implementation hypotheses at the first two Trusts in wave 1 over approximately 6 months. Key factors impacting on implementation in wave 1 will be identified and then discussed with stakeholders in the Trusts in wave 2. The research team will work with these stakeholders to collaboratively design implementation plan version 2 (IPv2) in these Trusts. Further data were collected to test the impact of this and to identify additional factors which are impacting on the longer term embedding of the intervention. A third iteration of the IP will be developed with staff in the wave 3 Trusts (IPv3). IPv3 will be introduced to the final Trusts at each site (wave 3), and data from this wave, combined with longitudinal data from the ongoing four Trusts from wave 1 and wave 2, will inform the final draft (IPv4).

It is difficult to anticipate the ideal number of iterations to develop an implementation plan, but we have proposed three waves, providing in-depth data from six NHS Trusts as our research experience suggests this will provide sufficient depth of understanding across a range of different settings, whilst ensuring the data collection and analysis is manageable within the timeframe and resources provided for the study.

#### Phase 3—finalising the implementation plan

We will synthesise data across all Trusts to develop a national implementation plan for REACT. The exact nature of the implementation plan cannot be determined at this stage as it will depend on study findings, but we anticipate it may include a video rationale for the use of REACT including research and policy context, a step-by-step guide to successful implementation of REACT, online staff training toolkit, a summary of resources needed to implement REACT, measures/evaluation tools to evaluate uptake and outcome for relatives, and case examples describing the process of implementation across participating Trusts—where the focus is on identifying and overcoming key barriers.

Wider applicability of the implementation plan for REACT will be tested by presenting the data to key stakeholders from non-participating Trusts and inviting them to consider challenges associated with delivering this within their Trusts. This could lead to additional edits to ensure our final implementation plan can be used across a wide range of mental health services.

Finally, we will take the findings from this study to other experts in the field of implementation science and consider how this data informs the implementation of other web-based supported self-management interventions across the NHS and further evolution of implementation theories.

### Data

Data collection will require a mixed methods approach. All methods have limitations, but used together, they strengthen the validity of the findings [54]. At each site, the following data will be collected to address each of the study objectives.

Data to identify the critical factors impacting on implementation of REACT (objective 2) will be primarily qualitative and will consist of:

1. Stakeholder reference groups (SRGs). These take place within each Trust at key points throughout the study. They allow the research team, Trust staff, and relatives to discuss how best to access data to help test the implementation hypotheses and how to use this data to revise the implementation plan. They are used flexibly throughout the study to ensure a collaborative approach, and both facilitate the research process, and contribute important data to the study.
2. Interviews with key stakeholders (commissioners, managers, frontline clinical staff, relatives, and service users). Semi-structured interviews will be conducted face to face (preferably) or by phone/skype if needed. The topic guide will focus on testing our implementation hypotheses whilst also allowing new ideas to emerge. Based on past experience [55], we anticipate conducting approximately 20 interviews at each Trust. All interviews will be transcribed in full and coded using NVivo software to aid data management.
3. Document analysis. Documents are most likely to provide data relevant to the context in which REACT is being implemented, but are likely to also cast light on the facilitation process. Examples of potentially relevant documents include national clinical audit reports [51], clinical care pathways, Trust carers strategy, Trust website and service user information leaflets, and service user and carer feedback. Interpretation of the contextual data from individual Trusts will be helped by comparison to nationally available data where possible, including national audit data. This will allow relative comparison of caseloads, referral rates, and duration of untreated psychosis (DUP). Document analysis will be used strategically to support the interview data.
4. Observation of naturally occurring meetings will be recorded using proformas developed for the study and designed to capture the relevant information to test our hypotheses in each context. Examples of relevant meetings may include Trust board meeting, adult mental health quality and performance meetings, CQUIN (Commissioning for Quality and Innovation) target strategy, EIP service business and clinical meetings, Carer’s strategy meetings, and PPI (public patient involvement) strategy meetings. Meetings will be strategically selected and informed by our interview and reference group data.

The selection of data sources will be informed by the specific hypotheses being tested, i.e. we will seek out data which is best placed to help us test our hypotheses.

Quantitative measures will be used to assess the uptake and use of REACT by NHS EIS teams and relatives (objective 1), investigate the impact of REACT on self-reported relatives outcomes at each site (objective 4), and investigate the resources (and costs) needed for successful implementation of REACT (objective 3). Specifically, we will assess the number of REACT accounts created for relatives to access the REACT site and the level of use of each module using web analytic statistics. We will collect basic demographic information about all relatives using REACT so we can better understand who uses it. Relatives who choose to visit the site will also be invited to take part in the collection of outcome data. Following online consent, they will complete questionnaires at baseline and again after 12 and 24 weeks to assess levels of distress (General Health Questionnaire-28; GHQ [56], wellbeing (Carer Wellbeing and Support Questionnaire [57]), quality of life (EQ-5D-5L [58]), and eHealth literacy (eHEALS [59]), and collect information about their caring role. These measures have been shown to be acceptable and sensitive to change [33]. Those who decline to complete the outcome measures can still receive the REACT intervention and contribute anonymously to the implementation data. No individual personal data will be available for relatives who declined or were not offered the toolkit. All data from relatives will be downloaded to a database held at the Liverpool CTRC.

Resources needed to deliver REACT will be identified using proformas designed specifically for this study. We will generate a list of the likely resources involved as part of Phase 1 and design measures to record this at each site. The proformas will be flexible to accommodate any additional resources identified during data collection but will include staff time, technology costs, and training or promotional materials.

### Analysis

Consistent with the case series design, data will first be analysed and presented within each Trust, before attempting to analyse similarities and differences between Trusts and how these can be explained. Where Trusts are divided into distinct clinical teams, these will be treated as embedded units, and where appropriate, similarities and differences between units will be investigated.

Qualitative data will be analysed using framework analysis, a pragmatic approach useful for applied research in which data is synthesised from different sources [60]. Our initial framework will be derived from both an initial process of familiarisation with the data and informed by our programme theory. We will use the framework flexibly, recognising that emergent data may inform further development of the framework. Reliability will be enhanced by use of regular data review meetings and a regularly updated codebook used across all Trusts. We will seek alternative interpretations of the data to maximise the validity of our findings. This analysis will inform our final report of the factors impacting on implementation.

In addition to this detailed data analysis, we will need to be able to continuously make sense of the data as it is collected, in order to develop our iterative implementation plans within a timeframe that suits the project and the needs of the NHS Trusts. This requires a more agile sense making at a “bigger picture” level to develop, implement, and evaluate each iteration of the implementation plan. As far as possible, this will be linked to specific data sources (interviews, workshops, documents, and meeting observations), but will not require a full thematic analysis or coding of transcribed data. Regular multi-disciplinary research team meetings and SRGs within the Trusts will ensure this remains grounded in the data.

Quantitative data will be analysed via descriptive statistics to summarise use of REACT at each Trust and overall, by staff and relatives. Descriptive statistics supported by graphical representation will be used to compare relatives’ outcomes over time (baseline, 12 and 24 weeks) within and across Trusts. Across the six Trusts, measures of use of the REACT toolkit will be explored as mediators of change over time.

The specific hypotheses generated in phase 1 will be tested by integrating both the qualitative and quantitative data.

### Public patient involvement

Relevant stakeholders are involved in IMPART at all stages. REACT was developed with extensive input from relatives and clinicians over many years [33, 61]. The team that developed the IMPART study design includes a relative of someone with a long history of psychosis and Director of the McPin Foundation that works to promote service users and relatives in mental health research. We have employed relatives to input to key aspects of the study including taking part in our monthly project management group (PMG), co-facilitating SRGs within each Trust, analysing qualitative data within each Trust, and developing each iteration of the implementation plan. NHS staff members are also key stakeholders and sit on the PMG, SRGs, and data analysis groups. We believe that involving relatives and staff will improve the delivery of the project, the experience of relatives in the research process, and how effectively the findings are disseminated.

### Project oversight

A Study Steering Committee (SSC) will provide oversight on behalf of the Project Sponsor (Lancaster University) and Project Funder (National Institute of Health Research) and ensure that the project is compliant with the Department of Health’s Research Governance Framework for Health and Social Care and the Guidelines for Good Clinical Practice. Members of the SSC include experts in implementation research, mental health, and service user/relatives from mental health services.

## Discussion

This study is designed to examine the factors impacting on implementation of a web-based intervention in a real world clinical setting. Participating NHS teams will be given access to the REACT toolkit and iterative versions of the implementation plan, allowing us to see in real time what influences use of REACT and how the implementation plan impacts on these factors. Thus, the findings will have high external validity and should be generalizable to other NHS Trusts and other web-based interventions, as well as informing further development of implementation theories.

However, inherent in the design, there are also a number of challenges we need to address. The first is the dual role of the research team as both observers of the implementation process to identify key factors impacting on this, and co-developers of the implementation plan, designed to address these factors. We acknowledge the problems inherent in this dual role, but believe that without this involvement, it would be impossible to understand the implementation process in the depth and detail required. The well-known Hawthorne effect [62] predicts that our attempts to capture data by setting up interviews, observing meetings, etc., are all likely to stimulate staff to find out more about REACT, check the online site, and possibly even invite relatives. To address this, we will ensure that our data collection is transparent and clear records are kept of the actions of the research team in facilitating implementation so that this can be incorporated into the analysis. In repeatedly taking the data back to the broader SRGs at each site and at key points throughout the study, we will ensure sufficient critical distance on our interpretation of all of the data, including our own role in the implementation process. In addition, the interpretation of the coded data will be checked at regular intervals in consultation with the wider research team who are able to question and identify where interpretations may reflect researcher bias.

Related to this dual role, our second challenge is to resist the urge to facilitate implementation of REACT at each Trust during the data collection phases. Our team have invested time and energy in developing REACT and as such are inherently motivated to see it successfully taken up in the NHS. The temptation is to try to immediately address barriers without allowing the natural implementation process to unfold and a deeper understanding of the implementation process to unfold. We are very aware of the need to watch and learn before developing the implementation plan, and our iterative design is intended to facilitate this approach. Research staff collecting data will maintain a reflexive diary to record their experiences in the field and reflect on how their role in data collection is also impacting on implementation.

The third challenge is to conduct an in-depth and thorough analysis of multiple sources of data collected across six geographically diverse NHS Trusts within a timeframe that also allows us to co-develop and deliver three iterations of the implementation plan. We will do this using two levels of analysis as outlined above; first a more detailed framework analysis which will give an in-depth understanding of the factors impacting on implementation of a web-based intervention to support relatives; and secondly a “bigger picture” analysis drawing on all data sources, our experience during the study, and staff and relatives in each of the participating Trusts that can be used in an agile way. This ensures our analysis is fit for purpose: the purpose of the in-depth analysis being to inform our theoretical understanding of the implementation of digital health interventions healthcare settings, and the second more pragmatic analysis to inform a series of iterative implementation plans which can be developed and tested in the 30 months’ timeframe of the study.

## Abbreviations

Apps: Applications
CQUIN: Commissioning for Quality and Innovation
CTRC: Clinical Trials Research Centre
DUP: Duration of untreated psychosis
eHEALS: The eHealth Literacy Scale
EIP: Early intervention in psychosis
EQ-5D-5L: European Quality of Life-5 Dimensions
GHQ: General Health Questionnaire
IMPART: Implementation of a Relatives’ Toolkit
IPv1: Implementation plan version 1
IPv2: Implementation plan version 2
IPv3: Implementation plan version 3
IPv4: Implementation plan version 4
IT: Information technology
NHS: National Health Service
NICE: The National Institute for Health and Care Excellence
NPM: Normalisation Process Model
NPT: Normalisation process theory
PMG: Project management group
PPI: Public patient involvement
REACT: Relatives’ Education And Coping Toolkit
SRGs: Stakeholder reference groups
SSC: Study Steering Committee
StaRI: Standards for Reporting Implementation Studies
UCL: University College London
UK: United Kingdom
US: United States
WL: Waitlist
